# Simple water infusion via a non-traumatic tube facilitates endoscopic resection of an appendiceal-orifice polyp

**DOI:** 10.1055/a-2764-4874

**Published:** 2026-01-13

**Authors:** Hajime Yoshii, Kazunori Takada, Kenichiro Imai, Sayo Ito, Kinichi Hotta, Hiroyuki Ono

**Affiliations:** 1 38471 Division of Endoscopy, Shizuoka Cancer Center, Shizuoka, Japan

**Fig. 1:**
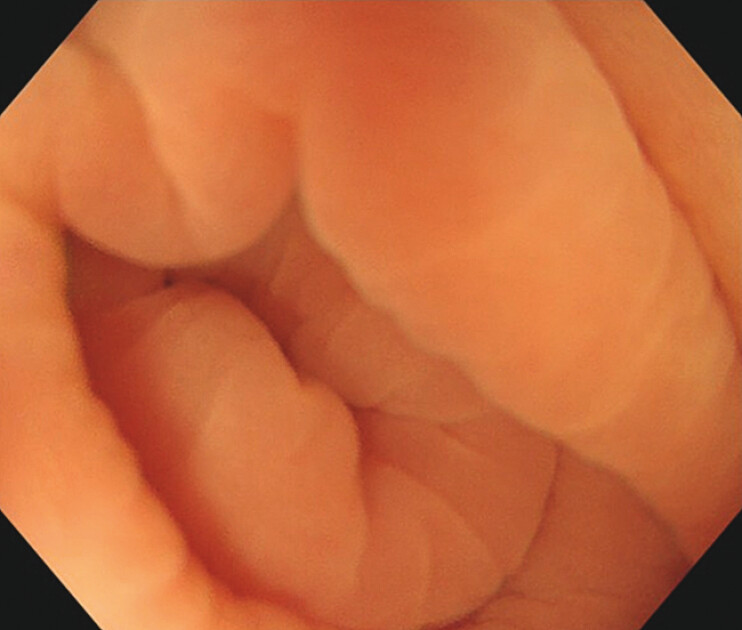
The lesion could not be visualized with water immersion.

**Fig. 2:**
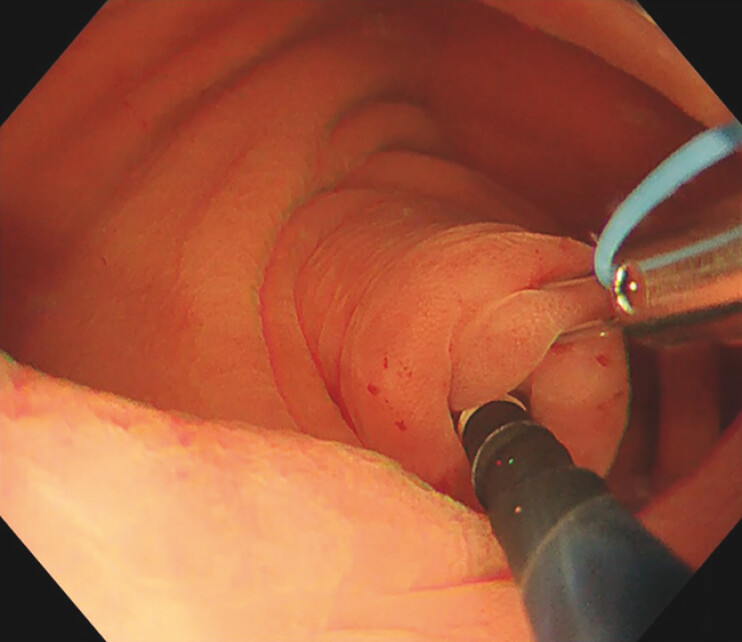
A non-traumatic tube was inserted into the appendiceal lumen.

**Fig. 3:**
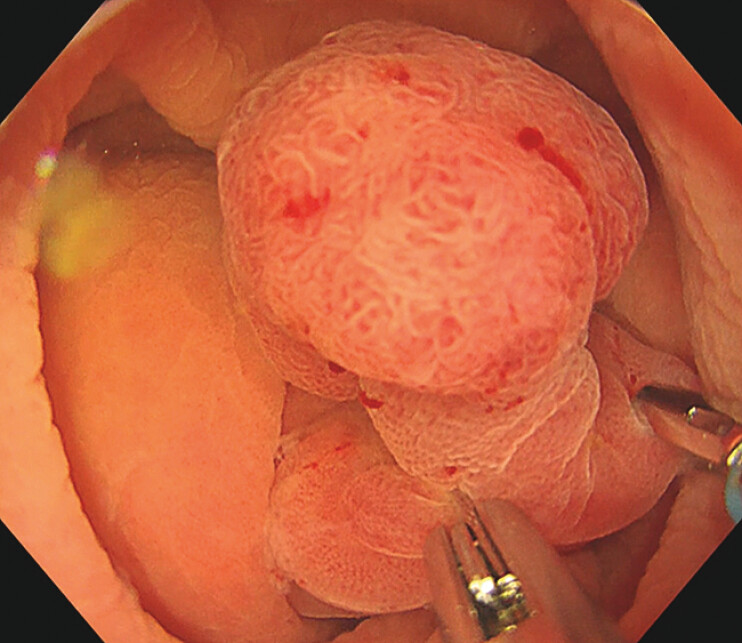
Continuous water infusion through the tube generated hydraulic pressure that extruded the polyp into the cecal lumen.

**Fig. 4:**
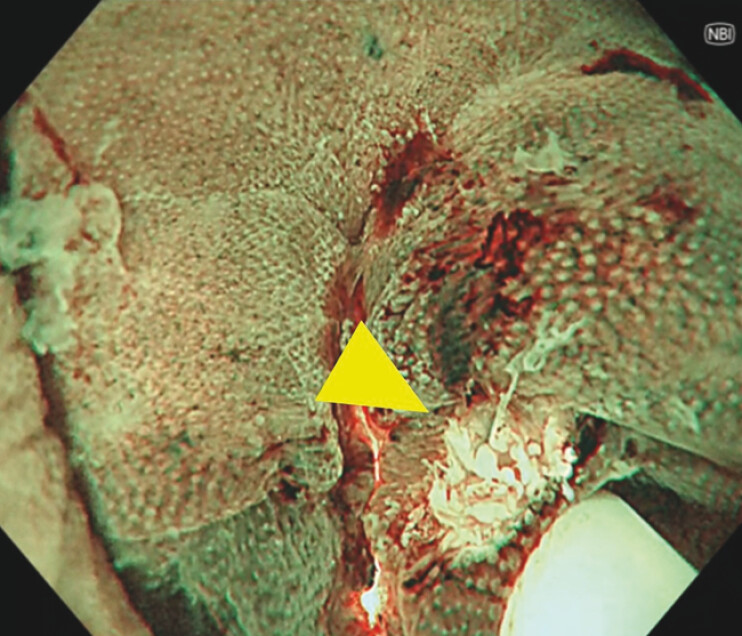
Complete resection was confirmed endoscopically. The arrowhead indicates the edge of the resection base.

**Fig. 5:**
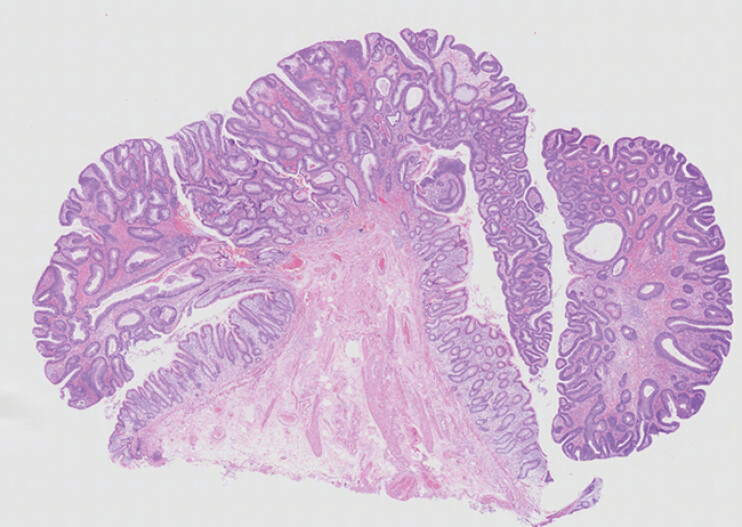
Histopathology revealed tubular adenoma.

We present the case of a 53-year-old man who was referred after fecal immunochemical test-positive colonoscopy revealing a polyp in the appendiceal orifice (AO). In our initial examination, the polyp could not be visualized with air insufflation or water immersion ( Fig. 1 ), even with traction using biopsy forceps. A repeat colonoscopy 3 months later failed to expose the lesion, even with traction using a multi-loop traction device (Boston Scientific Co. Ltd, Tokyo, Japan). Subsequently, a non-traumatic tube was carefully inserted into the appendiceal lumen ( Fig. 2 ). Continuous water infusion through the tube generated hydraulic pressure that extruded the polyp into the cecal lumen, thus permitting stable visualization ( Fig. 3 ). The lesion appeared pedunculated with a 10-mm head. Magnifying narrow band imaging revealed Japan NBI Expert Team classification Type 2A, consistent with adenoma. After placement of a hemostatic clip at the stalk base, en bloc resection was performed using underwater endoscopic mucosal resection. Complete resection was confirmed endoscopically and additional prophylactic clips were applied ( Fig. 4 ). The procedure was completed without any adverse events. Histopathological examination revealed a tubular adenoma with negative margins ( Fig. 5 ).

Polyps at the AO often require surgery because of difficulties in visualization and access 1 . To the best of our knowledge, this is the first report of targeted water infusion into the appendix via a non-traumatic tube to expose an AO polyp and enable safe resection using standard tools ( Video 1 ). The safety of appendiceal intubation and irrigation is supported by reports of endoscopic retrograde appendicitis therapy 2 3 . This simple and reproducible maneuver may expand endoscopic options and help avoid surgery for AO polyps.

Simple water infusion via a non-traumatic tube facilitates endoscopic resection of an appendiceal-orifice polyp.Video 1

Endoscopy_UCTN_Code_TTT_1AQ_2AD_3AB
